# Beyond the Liver: A Unique Case of Pyogenic Liver Abscess Caused by Peptostreptococcus micros Penetrating the Chest Wall

**DOI:** 10.7759/cureus.64177

**Published:** 2024-07-09

**Authors:** Giovannie Isaac-Coss, Alex Chow, Madhavi Reddy, Vikash Kumar, Mohammad Nawaz

**Affiliations:** 1 Internal Medicine, The Brooklyn Hospital Center, New York, USA; 2 Infectious Diseases, State University of New York (SUNY) Downstate Health Sciences University, New York, USA; 3 Gastroenterology and Hepatology, The Brooklyn Hospital Center, New York, USA; 4 Internal medicine, Jinnah Sindh Medical University, Karachi, PAK; 5 Internal Medicine, The Brooklyn Hospital Center, Brooklyn, USA

**Keywords:** percutaneous drainage hepatic abscess, hepatic abscess penetrating chest wall, peptostreptococcus micros, surgical drainage hepatic abscess, parvimonas micros, management hepatic abscess, p. micra, liver abscess drainage, pyogenic hepatic abscess, pyogenic liver abscess (pla)

## Abstract

Liver abscesses are uncommon pyogenic infections with diverse microbiology, often involving enteric gram-negative bacilli such as *Escherichia coli *and *Klebsiella pneumoniae*. Standard management includes antibiotic therapy and abscess drainage. We present a case of a 37-year-old male with chronic right upper quadrant abdominal pain, who was found to have an enlarging liver mass infiltrating the chest wall and right-side chest ribs, ultimately diagnosed as a large pyogenic liver abscess (PLA) extending into the chest wall. Notably, the abscess was attributed to *Peptostreptococcus micros*, a rarely isolated pathogen in liver abscesses. Despite initial unsuccessful percutaneous drainage, surgical intervention proved necessary for definitive treatment. This case underscores the diagnostic challenge posed by uncommon pathogens in liver abscesses and emphasizes the effectiveness of surgical drainage in managing refractory cases.

## Introduction

Pyogenic liver abscesses (PLAs) are infections that involve enclosed suppurative material within the liver parenchyma. They are the most common type of visceral abscesses and makes up about 13% of intra-abdominal abscesses, according to some reports [1]. Prevalence can vary depending on geographic location. In the United States, reports show an annual incidence of 2.3 cases per 100,000 people [2]. Reports indicate a much higher incidence in Asia, with a yearly incidence of up to 17.6 cases per 100,000 people [3]. Major risk factors include diabetes mellitus, pre-existing hepatobiliary disease, and liver transplant status. There is also a significant association between PLA and colorectal cancer, seen primarily in Asia. A pyogenic liver abscess usually develops from portal vein pyemia or ascending infection from the biliary tract. Another major route of infection is hematogenous seeding from the systemic circulation, especially when the abscess is due to streptococcal or staphylococcal species.

The microbiology of PLA is highly variable, with many pathogens described and demographics playing a significant role in determining the likely causative organisms [2]. Most abscesses are polymicrobial, with enteric gram-negative bacilli *Escherichia coli *and *Klebsiella pneumoniae* being among the most common. Anaerobes are often present, though they are often under-reported due to difficulties in culturing and characterization. Streptococcal species, especially the *Streptococcus milleri* group, and staphylococcal species are also commonly reported in PLA, particularly in Western countries [4]. Amebic and fungal abscesses have also been reported in the liver and are sometimes difficult to clinically differentiate from bacterial abscesses. Antibiotic treatment can be challenging given the variable microbiology.

Management of PLA involves a combination of antibiotics and drainage. Empiric antibiotics should cover streptococci, enteric gram-negative bacilli, and anaerobes. Coverage should also generally include *Entamoeba histolytica *until the causative pathogen(s) is identified or an amebic abscess is excluded. Common empiric regimens usually involve a third-generation or higher cephalosporin plus metronidazole. A fluoroquinolone with metronidazole is a common alternative. Therapy is usually then tailored to the isolated pathogen(s). Antibiotics should always be accompanied by drainage when feasible. Percutaneous drainage has become the preferred method of intervention due to general improvements in expertise, its less invasive nature, and usually favorable outcomes [5]. When performing percutaneous drainage, needle aspiration, and catheter drainage are the most commonly used interventions. Abscess size and complexity are significant factors in determining the type of intervention, with larger and more complex abscesses usually necessitating catheter drainage. Surgical drainage is rarely used as the primary intervention but remains an option when the patient fails percutaneous intervention or has a condition that necessitates surgical intervention.

## Case presentation

A 37-year-old male with a history of generalized anxiety disorder and panic attacks presented to the emergency room for an enlarged liver mass invading the abdominal and chest walls. Symptoms started about two to three months prior with sharp pain around the right upper abdomen and right lower chest wall. He initially presented to a different emergency room, where a chest x-ray was negative for acute findings. He was diagnosed with costochondritis and discharged with non-steroidal anti-inflammatory drugs. However, his pain did not improve. Over the next few months, the patient developed new symptoms, including night sweats, 15 lb unintentional weight loss, fatigue, loss of appetite, dry cough, back pain, and constipation. His primary care provider ordered an MRI of the abdomen, which showed multiple liver lesions concerning for malignancy. He was referred to oncology, where a PET/CT was performed, showing a multilobulated liver mass with a significantly hypermetabolic thickened wall encasing multiple right ribs, concerning for liver embryonic sarcoma and hypermetabolic portocaval lymph nodes. He was then referred to the emergency department for further evaluation. On social history, he denied any drug use, alcohol, or smoking. He admitted having a dog with an unspecified skin condition. He was born and raised in the United States. No recent travel history. The patient otherwise denied fevers, chills, nausea, vomiting, diarrhea, or dysuria.

**Figure 1:**
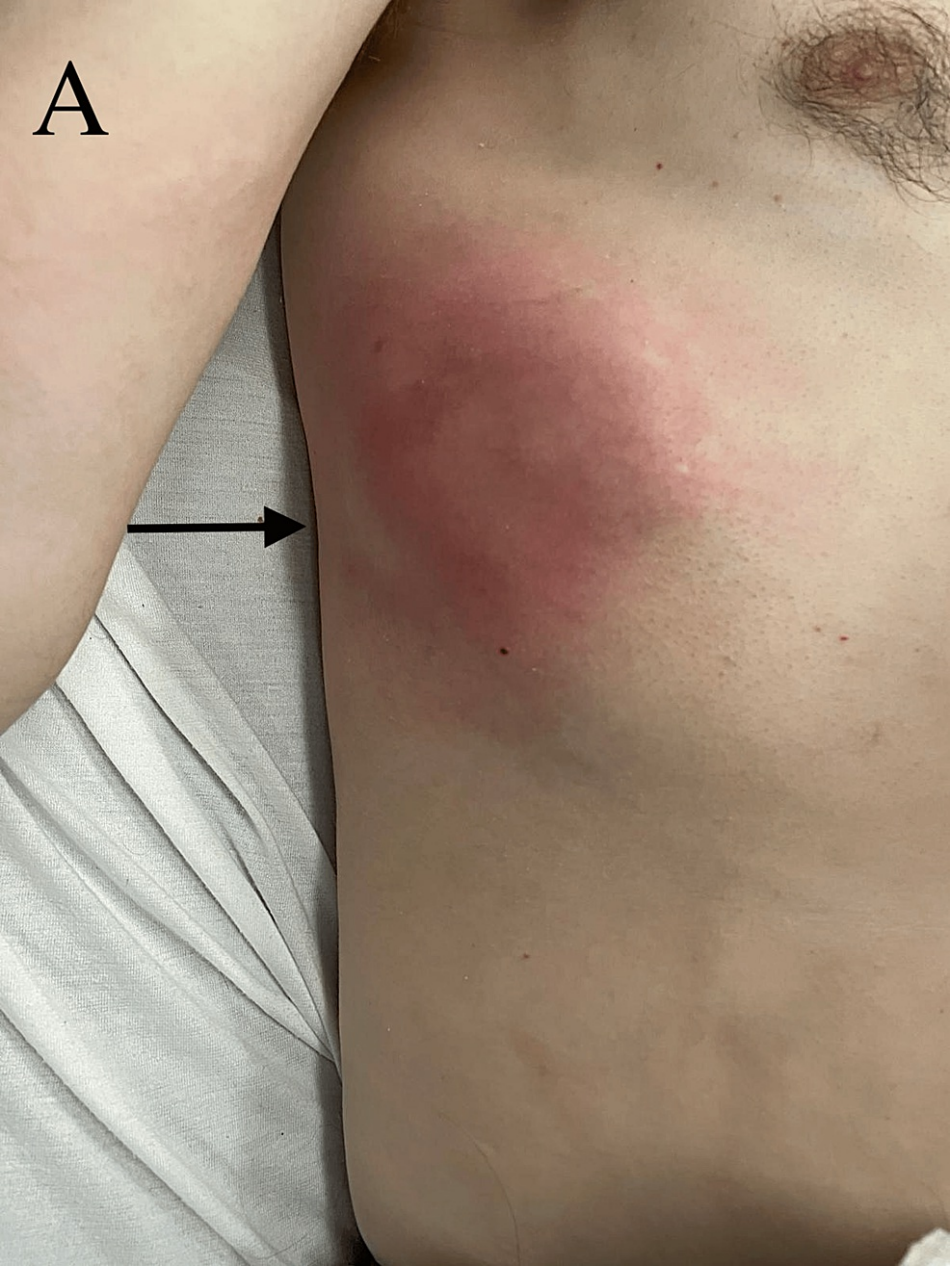
Physical examination. A: Physical examination showing an 11 x 10 x 15 cm erythematous tender irregular solid fixated lesion with a round contour involving the right lower chest area across several ribs.

On presentation, the patient was afebrile. The physical exam was remarkable for an erythematous circular protrusion on the right lower chest wall and mild abdominal tenderness (Figure 1).

**Figure 2:**
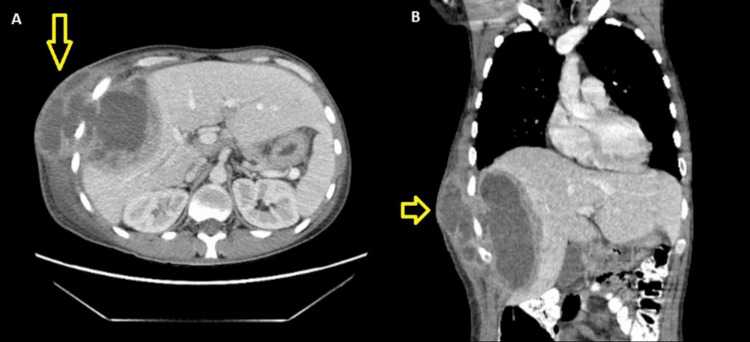
CT chest/abdomen/pelvis showing a large 10 x 7 x 15 cm hypodense collection with multiple enhancing septa with enhancing capsule (yellow arrow). There is mild surrounding hyperenhancement of the liver parenchyma, suggesting inflammation. Extension of complex collection abscesses to the right lateral chest wall through the right 7th through 9th intercostal rib spaces. A: Sagittal view; B: Coronal view.

Laboratory workup was significant for leukocytosis of 16 K/cmm. Initially, antibiotics were not started, but the patient became febrile with a temperature of 102.8°F, and the leukocytosis increased to 21.9 K/cmm. Antibiotic therapy with vancomycin, cefepime, and metronidazole was started for broad-spectrum coverage. A CT scan of the abdomen and pelvis with contrast showed a large 10 x 7 x 15 cm hypodense collection with multiple enhancing septa and an enhancing capsule extending through the right lateral chest wall (Figure 2).

Following concern for malignancy, AFP, CA 19-9, and CEA were ordered; however, they all came back within normal limits. Blood cultures remained negative since admission, and HIV screening was negative. The hepatitis panel showed Hepatitis B immunity, Hepatitis A non-immune, and Hepatitis C antibody non-reactive; *Entamoeba* and *Echinococcus *serologies were negative (Table 1). 

**Table 1:** Relevant laboratory workup. H: High; L: Low; TB = Tuberculosis; IgG = Immunoglobulin G.

Relevant labs	Results	Reference
Complete blood count (CBC)	White blood cell count = 16 K/cmm (H)	(4.8–10.8 K/cmm)
	Hemoglobin = 8.4 g/dL (L)	(13.1–15.5 g/dL)
	Hematocrit = 27% (L)	(39%–47%)
	Platelets = 481 K/cmm	(130–400 K/cmm)
Basic metabolic panel (BMP)	Creatinine = 0.7 MG/DL	(0.7–1.3 MG/DL)
Hepatic function panel	Bilirubin total = 0.3 MG/DL	(0.2–1.2 MG/DL)
	Bilirubin direct = 0.1 MG/DL	(0.0–0.5 MG/DL)
	Aspartate aminotransferase = 15 U/L	(8–34 U/L)
	Alanine transaminase = 14 U/L	(6–55 U/L)
	Albumin = 3.1 (L)	(3.5–5.0 G/DL)
	Alkaline phosphatase = 123 U/L	(40–150 U/L)
	Total protein serum = 6.9 G/DL	(6.4–8.3 G/DL)
Acute hepatitis panel	Hepatitis B surface antibody = reactive	Non-reactive
	Hepatitis B core antibody = non-reactive	Non-reactive
	Hepatitis A IgG antibody = non-reactive	Non-reactive
	Hepatitis B surface antigen = non-reactive	Non-reactive
	Hepatitis C antibody = non-reactive	Non-reactive
Microbiology	Blood cultures = no growth	No growth
	Hepatic abscess fluid culture = Peptostreptococcus micros	No growth
Serology	Human immunodeficiency virus antigen/antibody = non-reactive	Non-reactive
	Alfa fetoprotein = 1.1 ng/ML	< 6.1 ng/mL
	Carcinoembryonic antigen = 1.6 ng/mL	(< = 3.0 ng/mL)
	Carbohydrate antigen 19-9 = 7.3 U/mL	(0.0–37.0 U/mL)
	Entamoeba histolytica antibody = negative	Negative
	Echinococcus antibody (IgG) = negative	Negative
	Quantiferon-TB = negative	Negative
Surgical pathology	Liver mass biopsy = rare benign fibrous stroma tissue lined by a single layer of ciliated columnar cells and microabscesses, favoring an inflammatory process	Normal liver tissue
	Fluid cytology = negative for malignant cells	Negative for malignant cells
Inflammatory markers	Erythrocyte sedimentation rate = 75 MM/HR (H)	(0–15 MM/HR)
	C-reactive protein = 211.31 mg/L (H)	(< 5.00 mg/L)
	Ferritin = 660 NG/ML (H)	(22–275 NG.ML)

**Figure 3:**
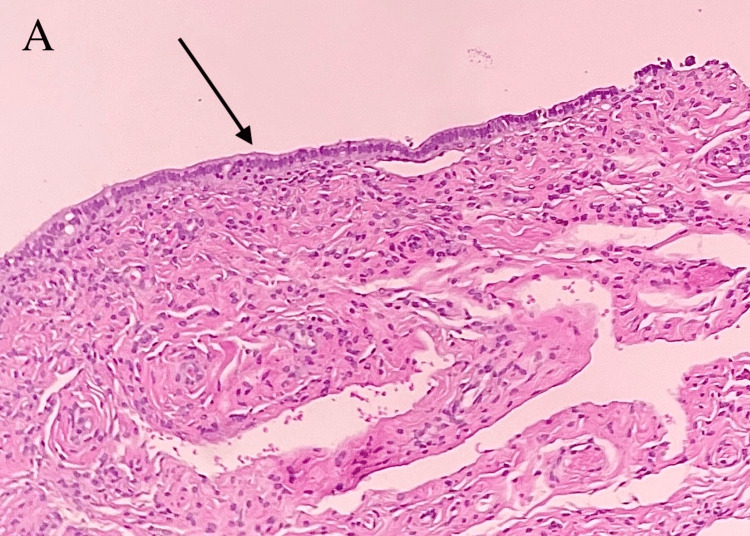
Surgical pathology; liver biopsy, microscopy. A: Medium power view (hematoxylin and eosin, 100X) showing viable ductal tissue with microabscesses (black arrow).

**Figure 4:**
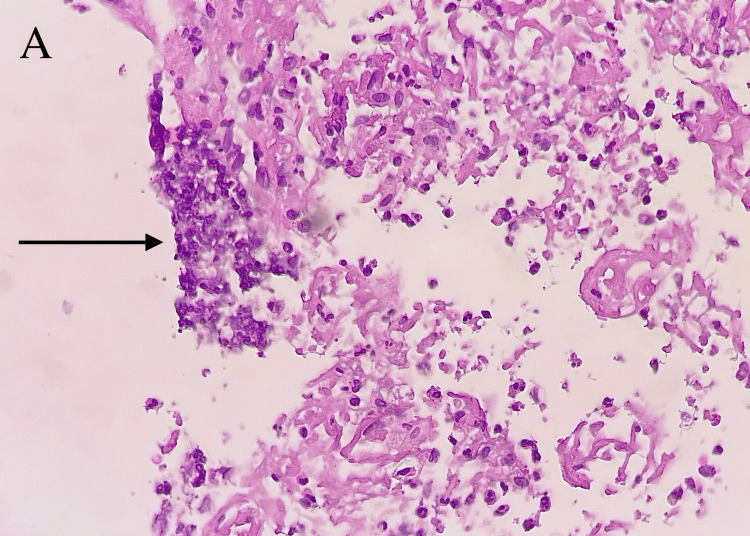
Surgical pathology; liver biopsy, microscopy. A: High power view (hematoxylin and eosin, 400X) showing necrotic tissue with acute inflammation (black arrow).

Interventional radiology (IR) was consulted, and the patient was taken for biopsy and drainage. Approximately 200 cc of thick, foul-smelling yellow fluid was drained and sent for analysis. The initial drainage culture showed no growth. The catheter drain was kept in place, but the patient was unable to tolerate it due to significant pain. Due to poor response to catheter drainage, surgery was consulted, and the patient underwent exploratory laparotomy with incision and drainage of the chest wall and liver abscess. An amount of 100 cc of pus was removed from both cavities. Traditional and fungal cultures were collected. The patient clinically improved rapidly after surgical drainage. All cultures remained with no growth but were held to isolate slow-growing and fastidious organisms such as *Actinomyces* and *Nocardia*. Cytology showed inflammatory cells consistent with an abscess, with no malignancy. Tissue biopsy showed rare benign fibrous stromal tissue lined by a single layer of ciliated columnar cells and microabscesses, favoring an inflammatory process (Figures 3, 4). 

Drain output ceased and the drains were removed. Antibiotics were stopped. The patient was discharged with follow-up in the surgery clinic. Following discharge, the IR drain culture grew *Peptostreptococcus micros (P. micros)*. The media used to culture the anaerobic organism included blood agar and phenylethyl alcohol blood agar. The surgical abscess culture showed no growth after 21 days. The patient was reassessed in the surgery clinic three days and one month after discharge. No additional antibiotics were started. The patient remained clinically improved and stable.

## Discussion

The case presents multiple rare and unique findings, including a PLA with chest wall invasion, a rare causative pathogen,*P. micros,* and an unusual management approach involving surgical intervention. This combination of findings has not been described previously in the literature.

Liver abscesses are generally confined to the liver. Abscess rupture is a rare complication, with a prevalence of around 3.8% [6]. Ruptures typically involve complications in the pleural and peritoneal spaces. Liver abscess invasion into the chest wall has rarely been described [7,8]. Given this unique presentation, malignancy was initially considered based on the original imaging findings. When chest wall involvement is described, the causative pathogens are usually atypical, including *Entamoeba* and *Mycobacterium tuberculosis*. Chest wall involvement draws similarities to empyema necessitans, a rare complication of empyema resulting in rupture/invasion of the chest wall. The causative pathogens in empyema necessitans are also usually atypical, including *Mycobacterium tuberculosis*, *Actinomyces* spp., and *Nocardia* [9]. Given these parallels, cultures collected for this case were prepared with these pathogens in mind. Due to the rarity of this presentation, empiric antibiotics were still directed at typical pathogens, including enteric gram-negative bacteria and anaerobes.

The microbe isolated in this case was *P. micros*. The taxonomy of this organism has evolved over the years. Most recently, this species was reassigned to *Parvimonas micra *in 2006. Our literature review includes both nomenclatures. Regardless of the naming convention, *P. micros* belong to the genus of gram-positive anaerobic cocci (GPAC). Other notable GPACs include *Finegoldia magna* (previously *Peptostreptococcus magnus* and other *Peptoniphilus *spp.). They are all commensal and usually non-pathogenic organisms that typically colonize the oropharynx, gastrointestinal, and genitourinary tracts [10]. Incidences of infections are likely underreported due to difficulty with specimen cultivation, often requiring prolonged incubation. Improvements in culture techniques and the wider use of molecular and advanced testing, such as 16S ribosomal sequencing and matrix-assisted laser desorption/ionization (MALDI-TOF), have increased the ability to detect these organisms. They have been described in a wide spectrum of infections, including skin and soft tissue, bloodstream, CNS, endocarditis, intra-abdominal, and female pelvic infections. Each species has a different predilection for causing various infections. Risk factors for infection include advanced age, malignancy, and immunodeficiency. *P. micros *is commonly associated with periodontal infections, though it has been reported to cause other infections [11-13]. It is usually a component of polymicrobial infections in most of these settings, though mono-infections have been described as well. Multiple cases of PLA with *P. micros *have been reported [14,15]. However, none describe a liver abscess invading the chest wall. This case expands the scope of infections that can be caused by *P. micros*.

This case also highlights an uncommon approach to managing PLAs involving surgical drainage. Most PLAs are managed via percutaneous drainage, either with needle aspiration for small, simple abscesses or catheter drainage for large, complex abscesses. Multiple randomized controlled trials have demonstrated better outcomes with catheter drainage compared to needle aspiration in the management of abscesses larger than 5 cm [16-18]. Surgical drainage is often reserved for patients with inadequate responses to percutaneous drainage or for abscesses not amenable to percutaneous drainage. As such, no randomized trial can ascertain its effectiveness. This patient qualified for surgical management due to the failure of percutaneous drainage, possibly due to the complexity of the abscess. Adequate source control was achieved with surgical management, and the patient rapidly improved clinically. Consequently, the need for extensive postoperative antibiotics was deferred. Short courses of antibiotics have been demonstrated to be appropriate when successful surgical source control is achieved, and the patient shows appropriate clinical improvement [19,20]. This patient remained clinically improved and stable over a month after discharge, demonstrating successful management.

## Conclusions

To summarize, this case highlights a rare complication of PLAs with chest wall invasion. The isolated pathogen was *Peptostreptococcus micros *(*P. micros*), a gram-positive anaerobic cocci commensal organism that has been shown to cause liver abscesses but never an abscess that invades the chest wall. The infection was successfully managed with surgical drainage and a shorter course of appropriate antibiotics. This is a novel combination of findings not previously reported in medical literature. Providers encountering a similar liver abscess in their practice should consider *P. micros* infection in their differential diagnosis, in addition to other gut gram-negative enterics and anaerobes, *Mycobacterium tuberculosis*, and *Entamoeba histolytica.* Cultures should be held for longer incubation to help cultivate causative pathogens. For a similar abscess that may not respond to percutaneous drainage, surgical drainage can be considered. Shorter courses of antibiotics may be justified if surgical drainage is successful.
